# Identification of Candidate Genes and Biosynthesis Pathways Related to Fertility Conversion by Wheat KTM3315A Transcriptome Profiling

**DOI:** 10.3389/fpls.2017.00449

**Published:** 2017-04-06

**Authors:** Jiali Ye, Yang Duan, Gan Hu, Xingxia Geng, Gaoming Zhang, Pengjiao Yan, Zihan Liu, Lingli Zhang, Xiyue Song

**Affiliations:** College of Agronomy, Northwest A&F UniversityYangling, China

**Keywords:** jasmonate, male fertility, MYB, phenylpropanoid biosynthesis, pollen development, RNA sequencing, wheat

## Abstract

The *Aegilops kotschyi* thermo-sensitive cytoplasmic male sterility (K-TCMS) system may facilitate hybrid wheat (*Triticum aestivum* L.) seed multiplication and production. The K-TCMS line is completely male sterile during the normal wheat-growing season, whereas its fertility can be restored in a high-temperature environment. To elucidate the molecular mechanisms responsible for male sterility/fertility conversion and candidate genes involved with pollen development in K-TCMS, we employed RNA-seq to sequence the transcriptomes of anthers from K-TCMS line KTM3315A during development under sterile and fertile conditions. We identified 16840 differentially expressed genes (DEGs) in different stages including15157 known genes (15135 nuclear genes and 22 plasmagenes) and 1683 novel genes. Bioinformatics analysis identified possible metabolic pathways involved with fertility based on KEGG pathway enrichment of the DEGs expressed in fertile and sterile plants. We found that most of the genes encoding key enzyme in the phenylpropanoid biosynthesis and jasmonate biosynthesis pathways were significant upregulated in uninucleate, binuclate or trinucleate stage, which both interact with MYB transcription factors, and that link between all play essential roles in fertility conversion. The relevant DEGs were verified by quantitative RT-PCR. Thus, we suggested that phenylpropanoid biosynthesis and jasmonate biosynthesis pathways were involved in fertility conversion of K-TCMS wheat. This will provide a new perspective and an effective foundation for the research of molecular mechanisms of fertility conversion of CMS wheat. Fertility conversion mechanism in thermo-sensitive cytoplasmic male sterile/fertile wheat involves the phenylpropanoid biosynthesis pathway, jasmonate biosynthesis pathway, and MYB transcription factors.

## Introduction

Heterosis is a widespread phenomenon in all plant species (Lippman and Zamir, 2007) and hybrid seed production has a major impact on crop improvement in order to meet the global demand for food. Cross-breeding can improve the crop quality as well as greatly increasing productivity (Darwin, 1876). The advantages of two-line systems, such as their easy application, lower cost, and simple restoration of fertility, have prompted wide attention and research since two-line high-yielding hybrid rice (He et al., 1992). Thermo-/photo-sensitive cytoplasmic male sterile lines can grow in environments below or above a critical temperature or photoperiod where their fertility will change in a critical period and thus male sterile plant fertility conversion occurs (Zhang et al., 2003). Therefore, thermo-/photo-sensitive cytoplasmic male sterile lines can self-fertilize without a maintainer line by sowing at different times or places, but they can also be used to produce hybrid seeds as female parents. Thus, these systems further simplify the seed production procedures during breeding, reduce the costs of seed production, improve the yield and seed purity, and they have great value during practical breeding.

KTM3315A is an *Aegilops kotschyi* cytoplasmic thermo-sensitive male sterile (K-TCMS) line, which has the short arm of chromosome 1B from *Triticum macha*, and it exhibits complete male sterility at normal wheat growth temperatures (<18°C). The sterility of KTM3315A is partially restored when plants are grown at temperatures above 20°C during the growth stages 45–52 (Song et al., 2013). Male fertility is not correlated with day length. KTM3315A performs well in practical hybrid wheat breeding and it can produce great quantities of seeds. Therefore, the TCMS-dependent two-line breeding system will probably be adopted widely in hybrid wheat production. The development of TCMS wheat lines demonstrates that there is great potential for producing hybrid wheat seed on a commercial scale.

Due to the development of high-throughput sequencing technology, transcriptome sequencing is now used widely. Recently, the transcriptome profiles of sterile lines and their fertility restorers have been investigated in various species during specific developmental stages, thereby demonstrating that a large number of differentially expressed genes (DEGs) participate in many metabolic pathways, e.g., in *Brassica napus* (An et al., 2014), cotton (Suzuki et al., 2013), cabbage (Wang et al., 2016), chili pepper (*Capsicum annuum*) (Liu et al., 2013), kenaf (Chen et al., 2014), watermelon (Rhee et al., 2015), radish (Mei et al., 2016), rice (Bai et al., 2015), and Welsh onion (Liu et al., 2016). However, the complexity of the wheat genome and the lack of complete reference genome mean that transcriptome sequencing in male sterile wheat remains difficult. There were few transcriptome sequencing of TCMS wheat, and the existing few cases were RNA-seq of chemical hybridization agent-induced wheat (Liu et al., 2014).

Male sterility in wheat has been investigated using cytological, physiological, and molecular biology techniques to understand the mechanism of pollen abortion. Before the introduction of high-throughput sequencing technology, two-dimensional gel electrophoresis (Li et al., 2011), DDRT-PCR (Zhao et al., 2007), SSH (Li et al., 2008), and cDNA-AFLP (Song et al., 2006) were employed to obtain the gene expression profiles related to male sterility in wheat. The mechanism of male sterility has also been studied with respect to polyubiquitin-related protein and DNA methylation (Ba et al., 2014; Liu et al., 2014). However, the transcription profiles of cytoplasmic male sterile wheat have been investigated little.

The phenylpropanoid biosynthesis pathway plays a vital role in plants, where it affects many important traits related to the synthesis of lignin, flavonoids, and other secondary substances. Phenylpropanoid biosynthesis is an energy-consuming and irreversible process, and many environmental factors affect phenylpropanoid biosynthesis, such as light, temperature, hormones, the circadian rhythm, stressful conditions, diseases, insect pests, and mechanical damage will affect its biosynthesis (Qin et al., 2014). Studies show that plant secondary metabolites are highly complex and the expression of relevant genes is strictly regulated in plant cells, specifically structural genes and regulatory genes. The structural genes directly encode several enzymes (such as PAL, C4H, and CHS) associated with phenylpropanoid biosynthesis (Ouyang and Xue, 1988), whereas the regulatory genes control the intensity and modality of structural gene expression. MYB transcription factors are important gene regulators, which are responsible for regulating plant growth and development (Craven-Bartle et al., 2013). Many studies have identified MYB transcription factors in different plant families and species that participate in phenylpropanoid biosynthesis (e.g., *Arabidopsis*, maize, petunia, and tobacco) (Craven-Bartle et al., 2013; Mu et al., 2015). Jasmonic acid and its derivatives are rapid signaling molecules that respond to stimulation, which have various physiological functions in plants. Jasmonates regulate the expression of defense genes and the biosynthesis of secondary metabolites by interacting with transcription factors (Qi et al., 2011). Many members of the MYB transcription factor family are involved in the jasmonic acid response, such as MYB21, MYB24, MYB32, MYB39, and MYB108. Jasmonates can induce phenylpropanoid biosynthesis in plant cells (Ma and Zhao, 2011), as well as being key signals that participate in mature pollen anther dehiscence (Qi et al., 2011). Thus, the jasmonate biosynthesis pathway and MYB transcription factors are closely related to pollen development and fertility via the phenylpropanoid biosynthesis pathway.

In this study, in order to insight into the molecular mechanisms and candidate genes related to the fertility conversion in K-TCMS wheat, RNA-seq was employed to sequence the transcriptomes of anthers from K-TCMS line KTM3315A, where bioinformatics analysis identified several candidate genes and showed that two important biosynthesis pathways interact with MYB transcription factors to affect fertility conversion.

## Materials and Methods

### Plant Materials

In October 2015, the K-type thermo-sensitive male-sterile wheat line KTM3315A was planted in pots. The pots were 30 cm high and 30 cm in diameter. Topsoil was used to fill these pots and 10 seeds of KTM3315A was then sown in each of the pots. The pots were planted at the Northwest A&F University experiment station, in Yangling (34°29′ N, 108°08′ E), China, and managed according to standard field wheat production practices. On November 1, 2015, a total of 20 pots of the same growth KTM3315A grown in the field were transferred into two growth chambers (10 pots/each) until the pollen production stage, which were programmed with a day/night period of 14 h/10 h and a light intensity of 10000 lux, with a day/night temperature of 17°C/15°C (sterile conditions) and 22°C/20°C (fertile conditions), respectively (Supplemental Figure S1). We designated KTM3315A under sterile and fertile conditions as sterile (AS) and fertile (AF) lines, respectively. Before anther, one spike of each plant was bagged, and used for fertility identification by seed setting frequent and pollen fertility, the same developmental anthers of the remaining spikes were mixed as sequence materials except for one anther in each spikelet used to check fertility in the final three stages (late uninucleate, binucleate, and trinucleate stages) from AF and AS separately, and frozen directly in liquid nitrogen before storing at -80°C in an ultra-low temperature freezer. In addition, anthers obtained from both AF and AS in five stages (tetrad, early uninucleate, late uninucleate, binucleate, and trinucleate stages) were fixed in formalin-acetic acid-alcohol for cytological observations.

### Phenotypic Characterization and Cytological Observations

Photographs of the plant materials were obtained using a Nikon E995 digital camera (Nikon, Tokyo, Japan) mounted on a Motic K400 dissecting microscope (Preiser Scientific, Louisville, KY, USA). Different anther developmental stages were identified by staining with 1% acetocarmine, and the chromosomes were analyzed by staining with 4′, 6-diamidino-2-phenylindole (DAPI). To evaluate the viability of mature pollen grains, dehiscent anthers from mature flowers were stained using I_2_-KI (1 g iodine and 3 g potassium iodide in 100 mL water) (Chang et al., 2009). The anthers and microspores were analyzed by scanning electron microscopy, as described by Zhang L.P. et al. (2007), and observed with a JSM-6360LV scanning electron microscope (JEOL, Tokyo, Japan). Anthers at various developmental stages were fixed, embedded, and stained for transmission electron microscopy, as described previously (Cheng et al., 2013). Observations and image capture were performed with a JEM-1230 transmission electron microscope (JEOL, Tokyo, Japan).

### RNA-seq Library Preparation and Sequencing

Anthers obtained from spikelets of 100 plants at the same development stages in AF and AS for RNA-seq, were mixed respectively because of small size anther and sampling difficulties when grown in artificial climate boxes. The total RNA was isolated using an RNAiso for Polysaccharide-rich Plant Tissue kit [Takara Biological Engineering (Dalian) Co. Ltd, China] according to the manufacturer’s protocol. RNA degradation and contamination, especially DNA contamination, were monitored on 1.5% agarose gels. The concentration and purity of RNA were measured using a NanoDrop 2000 Spectrophotometer (Thermo Fisher Scientific, Wilmington, DE, USA). RNA integrity was assessed using an RNA Nano 6000 Assay Kit for the Agilent Bioanalyzer 2100 System (Agilent Technologies, Santa Clara, CA, USA). Total RNA was treated with RNase-free DNase I to remove any DNA contamination before cDNA synthesis. First-strand cDNA was synthesized using random hexamer primers and reverse transcriptase. Second-strand cDNA synthesis was performed using DNA polymerase I and RNase H. The library preparations were sequenced using an Illumina Hiseq platform and paired-end reads were generated. Illumina sequencing was performed by Beijing Biomarker Biotechnology, Co., Ltd (China).

### Sequence Alignment

Before analyzing the data, it is essential to obtain clean data from the raw data by data filtering, as well as ensuring that the reads are sufficiently high quality and accurate for the subsequent analysis. To ensure strict quality control for the data, the following filtering approach was employed: (1) remove joint reads; and (2) remove low quality reads (remove the proportion of N with greater than 10% reads, and where the quality value Q ≤ 10 base number accounted for more than 50% of the total reads) (Shi et al., 2011). After applying quality control procedures to obtain high quality clean data, the data were aligned with the reference genome sequence MIPSv2.2 to map the data (MIPSv2.2 download address^1^).

### Biological Analysis of DEGs

Cuffdiff (v2.1.1) was used to calculate fragments per kilobase of transcript per million mapped reads (FPKMs) for the genes in each sample (Trapnell et al., 2010). FPKMs were calculated based on the lengths of the fragments and the read counts mapped to fragments. For samples without biological replicates, differential expression analysis based on two samples was performed using the EBseq R package (Leng et al., 2013). Q-value < 0.01 and |log2 (fold change)| > 1 were used as the threshold values for significantly different expression levels. Gene functions in the transcriptomes obtained from KTM3315A under two different conditions were annotated using the following databases: NCBI non-redundant protein sequences (Nr) (Deng et al., 2006); Pfam (a database of protein families) (Finn et al., 2014); Swiss-Prot (a manually annotated and reviewed protein sequence database); Kyoto Encyclopedia of Genes and Genomes (KEGG) (Kanehisa et al., 2004); and Gene Ontology (GO) (Ashburner et al., 2000). We analyzed the DEGs based on their functions. According to KEGG pathways, we prepared metabolic pathways by using Cytoscape (Shannon et al., 2003) to obtain an interaction network diagram. We employed OmicShare small tools^2^ to obtain a heatmap, where the threshold parameters were set as no rows and column clusters. The amino acid sequences were predicted using expasy^3^ and ProtParam^4^ was used to predict the physical and chemical properties. The NCBI website^5^ was used to predict structural domains and SWISS–MODEL^6^ for protein tertiary structure prediction.

### qRT-PCR Analysis

Primers for qRT-PCR were designed using Primer Primer 5.0 software (Primer, Canada) and verify by Primer-BLAST^7^. Primers synthesized by Sangon Biotech (Shanghai) Co., Ltd, China. The actin (GenBank: GQ339766.1) gene was used as reference. Sequence-specific primers used for qRT-PCR were list in Supplemental Table S2. qRT-PCR was performed on a QuantStudio^TM^ Real-Time PCR System (Applied Biosystems, USA) using 2× RealStar Green Power Mixture [GenStar BioSolutions (Beijing) Co., Ltd, China] under the following cycling parameters: 95°C for 30 s, followed by 40 cycles at 95°C for 5 s and 60°C for 30 s. Each reaction mixture was 25 μl containing 0.5 μl diluted cDNA and 0.5 μl of each primer, 2× RealStar Green Power Mixture 12.5 μl and ROX Reference Dye II (50×) 0.5 μl. All of the qRT-PCR analysis were performed with at least three replicates. Relative gene expression levels were calculated using the 2^-ΔΔCt^ method (Livak and Schmittgen, 2001).

## Results

### Phenotypic Characteristics and Cytological Observations

**FIGURE 1:**
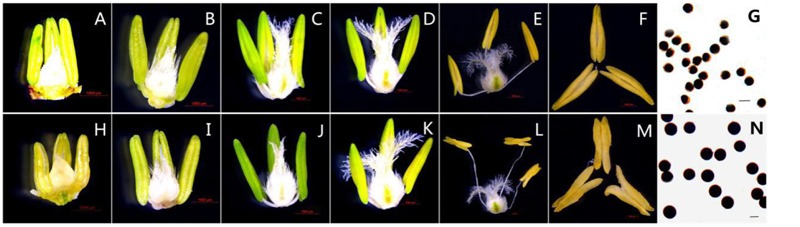
**Comparison of plant anther and pistil external morphological characteristics and I2-KI staining of KTM3315A under the different fertility condition (AS: **A–G**, AF: **H–N**). (A,H)** Tetrad stage; **(B,I)** early uninucleate stage; **(C,J)** the later uninucleate stage; **(D,K)** the binucleate stage; **(E–G,L–N)** the trinucleate stage. **(A–F,H–M)** Bar: 1000 μm; **(G,N)** Bar: 50 μm.

Based on morphological landmarks or cellular events observed by light microscopy and according to a previous classification of anther development (Zhang et al., 2013; Ba et al., 2014), we assigned wheat anther development to five stages. The results showed that the AS anthers appeared to be normal in the first few stages (**Figures 1A–D,H–K**), but there were some differences in anther morphology between AF and AS during the trinucleate stage. The AS anthers were light in color, empty, and flat. Anther wall cracking was not evident, and some sterile anthers were bent and tapered at the upper end, forked slightly at the base, and the anthers shed little or no pollen when mature (**Figures 1E,F**). By contrast, the AF anthers were fully plump and bright yellow. The upper and lower ends were slightly forked, with normal cracking and the shedding of loose powdery pollen (**Figures 1L,M**). Unlike the mature AF pollen, the AS pollen was not fully stained by 2% I_2_–KI and the seed-setting ratio was zero, which demonstrated that the pollen was abortive and the pollen abortion type was stainable abortion in AS, whereas the fertility was restored in AF that under high-temperature environment (**Figures 1G,N**).

**FIGURE 2:**
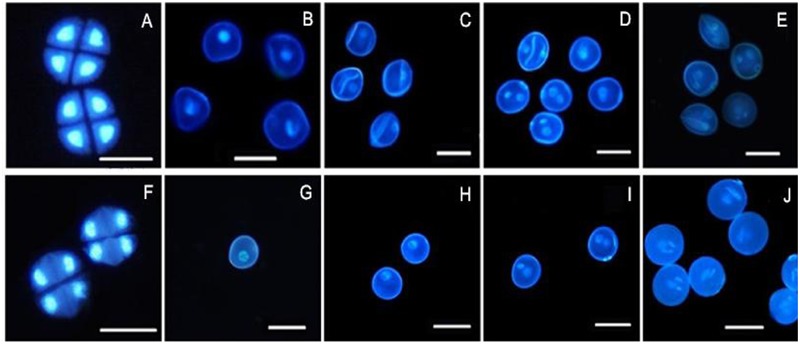
**Comparison of DAPI-stained of pollen of KTM3315A under the different fertility condition (AS: **A–E**, AF: **F–J**). (A,F)** Tetrad stage; **(B,G)** early uninucleate stage; **(C,H)** the later uninucleate stage; **(D,I)** the binucleate stage; **(E,J)** the trinucleate stage. Bar: 50 μm.

4′, 6-Diamidino-2-phenylindole staining is an effective method for observing chromosomal behavior and the growth of the nuclei. The DAPI staining results showed that the development of the chromosomes and the nuclei of sterile microspores exhibited abnormal development in AS where some cells had a wrinkled, condensed shape and folds could be observed in some cells, with nucleus malformation and asynchronous development until the trinucleate stage (**Figures 2A–E**). Microspore abnormalities were most significant in the late uninucleate stage in AS (**Figure 2C**). By contrast, fertile cells were normal, where the nucleus followed the normal development process to form mature pollen grains (**Figures 2F–J**). There were no evident differences between AF and AS in the tetrad stage, but in the later stages of sterile anther development, we clearly observed microspore contour deformity and abnormal nuclei, such as shrinkage and deformation of cellular structures in the late uninucleate and the binucleate stages, obvious differences in size between the vegetative nucleus and sperm nucleus, and normal nuclear cells could not be formed in the trinucleate stage (**Figure 2E**) because the sperm nucleus failed to form a spindle type, where it was round instead.

**FIGURE 3:**
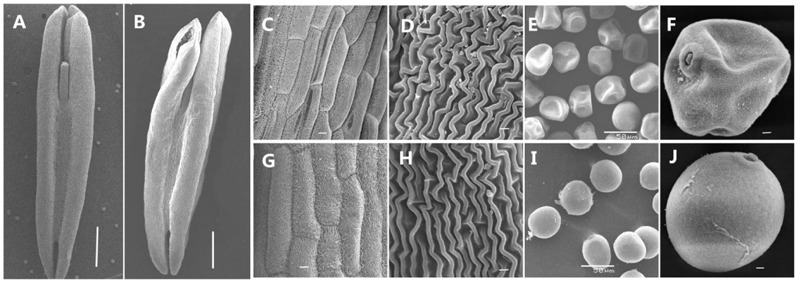
**Comparison of scanning electron micrograph observations in AS (A,C–F)** and AF **(B,G–J)** at the trinucleate stage. **(A,B)** Anther; **(C,D,G,H)** outer epidermal cells; **(E,F,I,J)** trinucleate cells. Scale bars are 1000 μm in **(A,B)**; 100 μm in **(C,G)**; 50 μm in **(E,I)**; and 30 μm in **(D,H,F,J)**.

To obtain a better understanding of the abnormalities in the AS anthers during the trinucleate stage, we used scanning electron microscopy to observe the outer epidermal surfaces of the anthers (**Figures 3A,B**), in AS which appeared to be smaller than those of the AF cells (**Figures 3C,G**) and they were more irregular in shape (**Figures 3D,H**). At the trinucleate stage, the fertile cells were rounded and plump, whereas the sterile cells appeared to be deformed and shrunken (**Figures 3E,F,I,J**). These results indicate that KTM3315A exhibited major impact on anther development and that male fertility conversion could be induced.

#### RNA-seq and Assessment of the Sequencing Results

To understand the molecular basis of fertility conversion in K-TCMS wheat, we performed high-throughput sequencing using six anther samples from AF and AS in the late uninucleate, binucleus, and trinucleate stages (**Figures 1C–E,H–L**). In total, 72.72 Gb of clean data were produced, with an average of 10.81 Gb clean data for each sample and the Q30 base percentage was ≥95.52% in each sample. All of the bases were distinguished, where the clean data GC content ranged from 53.89 to 55.06%. For each sample, the clean reads were aligned with a reference genome sequence, where the alignment efficiency ranged from 65.85 to 70.51% (Supplemental Table S1). After directly comparing the gene expression levels in different samples, we found that both the sequencing quality and gene expression level were generally identical (Supplemental Figure S2). Thus, the throughput and sequencing quality were sufficiently high to warrant further analysis.

**FIGURE 4:**
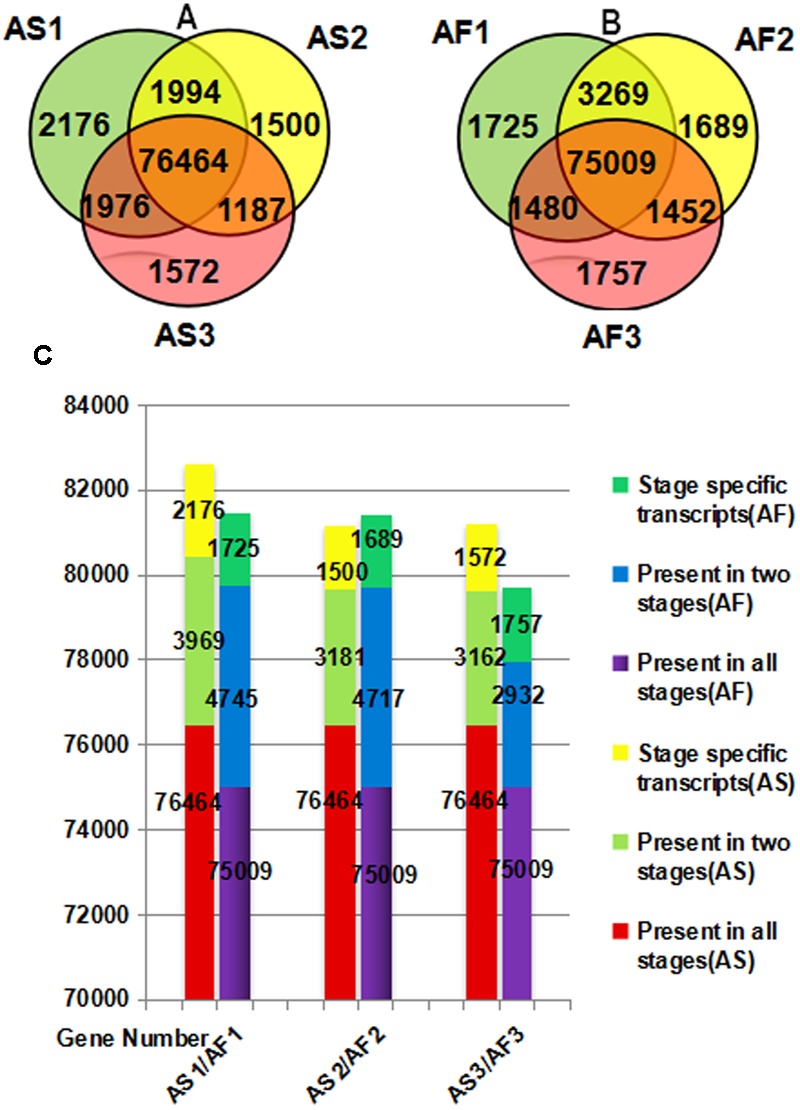
**Venn diagram (A,B)** and accumulation histogram **(C)** comparing the differentially expressed genes (DEGs) in AS and AF. The numbers of DEGs are shown in the different segments. **(A)** Total DEG genes in AS. **(B)** Total DEG genes in AF. **(C)** Accumulation histogram showing the transcriptome sizes during the three stages for AS and AF, as well as the changes in the numbers of transcribed genes during anther development in AS and AF. The horizontal axis indicates different samples and the vertical axis represents the number of genes.

There were differences in both the quality and quantity of gene expression among the three stages of anther development, which is a dynamic process. According to **Figures 4A,B**, specific genes were expressed during each stage in AS and AF, and the expression of some genes also occurred during one or two stages. We found that 2176 genes were specifically expressed during the late uninucleate stage in AS, which was significantly more than that in AF (1725). However, in the final two stages, more genes were specifically expressed in AF (1689/1757, binucleate/trinucleate) than AS (1500/1572). Using an accumulation histogram (**Figure 4C**), we clearly distinguished the general differences in transcription quantity among the developmental stages in AS and AF, as well as comparing the genes expressed in every stage. In AS, the number of genes expressed increased to 82609 during the late uninucleate stage, but 53 fewer genes were expressed in AS during the binucleate stage compared with the trinucleate stage. In AF, most genes were expressed in the late uninucleate stage with a total of 81479, followed by the binucleate stage with 81415, and the trinucleate stage with 79698. Excluding the binucleate stage, 270 more genes were expressed in AF than AS, and fewer genes were expressed in AF than AS in the other two stages. In both AS and AF, the co-expressed genes in the three stages exceeded 75000, where the numbers of co-expressed genes in two stages were 2923 to 4745, and the numbers of genes expressed specifically in only one stage were least. There were differences in fertility in AS and AF, which were attributable to the DEGs in each stage, but the overall level of gene expression did not differ greatly because the same type of material was used in different environments.

### Functional Classification of DEGs

**FIGURE 5:**
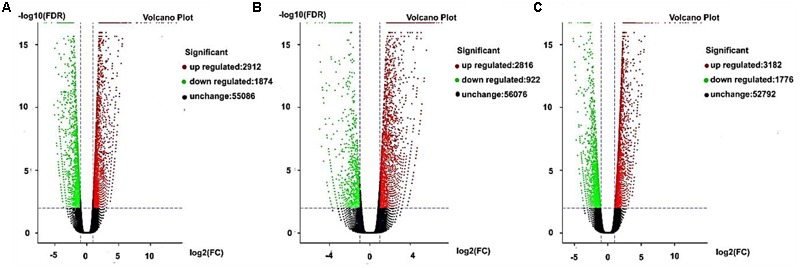
**Differentially expressed genes as volcano plots. (A–C)** Represent the expression levels of every gene in AF relative to AS during the later uninucleate, binucleate, and trinucleate stages, respectively. Every DEG point in the volcano plot represents one gene. The horizontal axis represents the difference in the specific gene expression levels in the two samples as a numerical value (ratio). The vertical axis shows the false discovery rate as negative values. When the absolute value of abscissa is higher, the relative difference in expression is greater between the two samples. A higher ordinate value indicates a more significant difference in expression, and thus the more reliable screening of DEGs. Green dots represent downregulated genes, red dots are upregulated genes, and black dots indicate no difference in the expression level in AF and AS.

Gene expression exhibits specificity in time and space, where external stimuli and the internal environment will affect the gene expression pattern. In this study, we detected DEGs based on fold changes of two times and FDR < 0.05 as the selection criteria. We used volcano charts to compare the DEGs in every stage for AF and AS based on significant differences (**Figures 5A–C**). In the late uninucleate, binucleate, and trinucleate stages, more than 5000 genes did not differ in their expression levels in both AF and AS. Some genes were upregulated or downregulated in AF compared with AS in every stage, and the number of upregulated genes in each stage was greater than the number of downregulated genes. The numbers of DEGs in the late uninucleate, binucleate, and trinucleate stages were 4786 (2912/1874, up-/ down-regulated), 3738 (2816/922), and 4958 (3182/1776), respectively, with a total of 13482 DEGs in the three stages (including duplicate genes in different stages). In general, the overall quantity of genes expressed differed slightly in the three stages, where some genes were expressed in a time-specific manner, but many were conserved.

In the three stages, the proportion of enriched secondary functions classified according to GO differed between AF and AS (Supplemental Figures S3A–C). However, there were no significant differences in the overall enrichment results for the binucleate stage compared with the other two stages. In terms of cellular components, the DEGs related to the extracellular matrix indicated that the information can be released to signal back to the cell, thus controlling patterns of gene expression and influencing cell fate (Brownlee and Berger, 1995). For biological processes, the DEGs related to circadian rhythm and cell death. In addition, DEGs associated with biological attachment and localization is also significantly different from background genes classified in this biological process. In terms of molecular functions, DEGs related to enzyme regulatory, antioxidant activities and guanyl-nucleotide exchange factor activity comprised a higher proportion relative to the total genes expressed, whereas fewer genes were related to protein binding transcription factor genes. Those DEGs have significant differences in the proportion of GO classification with the background genes will in command of programmed cell death, enzyme regulatory, antioxidant activities and guanyl-nucleotide exchange, and so on. This explains the difference of the antioxidant enzyme activity and the programmed cell death time between AF and AS, as well as the SNP (Supplemental Figures S4A,B).

**FIGURE 6:**
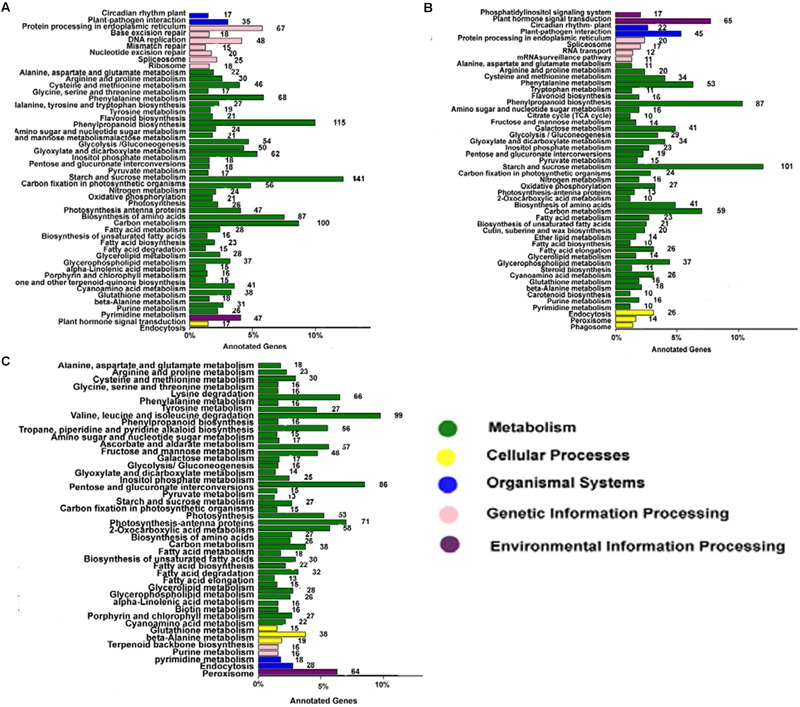
**KEGG classifications of DEGs.** The vertical axis shows the annotations of the KEGG metabolic pathways. The horizontal axis represents the gene numbers annotated in each pathway and the proportion relative to the total number of genes. **(A–C)** Represent the KEGG pathway classifications for DEGs in AF relative to AS in the late uninucleate, binucleate, and trinucleate stages, respectively. Each color represents a KEGG pathway and relationships are shown on the right-hand side.

Annotating the biological functions of DEGs helped to interpret the functions of the genes. Comparison of AF and AS in the late uninucleate, binucleate, and trinucleate stages showed that 1875, 1243, and 1654 DEGs, respectively, were annotated with KEGG pathway functions (**Figures 6A–C**). The DEGs in the three stages were enriched in nine pathways mainly, i.e., phenylpropanoid biosynthesis, circadian rhythm-plant, beta-alanine metabolism, starch and sucrose metabolism, cyanoamino acid metabolism, carbon metabolism, galactose metabolism, cysteine and methionine, and arginine and proline, as shown in Supplemental Table S3. These results were fairly consistent with the COG results (Supplemental Figure S5). For the starch and sucrose metabolism pathway, the numbers of DEGs were 141 in the uninucleate stage, 101 in the binucleate stage, and 86 in the trinucleate stage, where there was a decreasing trend. Most of the DEGs annotated in the first two stages were related to starch and sucrose metabolism because the main component of pollen is starch. Phenylpropanoid biosynthesis DEGs were also enriched greatly, with 115, 87, and 99 annotated DEGs in the late uninucleate, binucleate, and trinucleate stages, respectively. Many studies have shown that the phenylpropanoid biosynthesis pathway has an important role in plant male sterility according to transcriptome sequencing, but few have investigated the molecular mechanisms that allow this pathway to affect plant fertility.

### Identification of Transcription Factors Involved in Fertility Conversion

**FIGURE 7:**
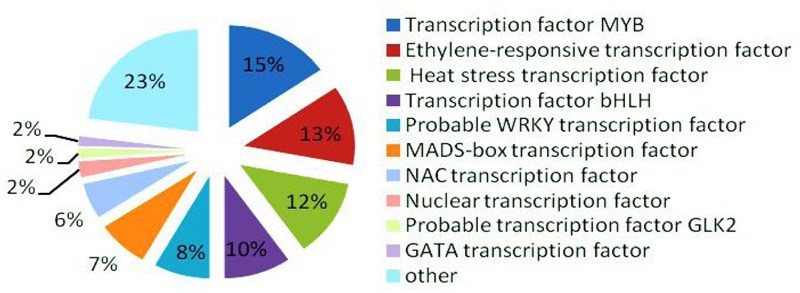
**Relative proportions of different transcription factors detected**.

In the late uninucleate, binucleate, and trinucleate stages, we annotated 3914, 3022, and 3919 DEGs, respectively, using Swiss-Prot^8^. The possible roles of these genes can be hypothesized according to the annotation information. In total, 318 DEGs were annotated as transcription factors among the 10855 Swiss-Prot genes, i.e., 49 MYB transcription factors (>15%), ethylene-responsive transcription factors (40), heat stress transcription factors (39), bHLH transcription factors (31), probable WRKY transcription factors (26), MADS-box transcription factors (24), NAC transcription factors (18), nuclear transcription factors (eight), probable GLK2 transcription factors (five), and GATA transcription factors (five) (**Figure 7**). MYB transcription factors comprised the highest proportion, so we suggest that they may have important roles in fertility decisions.

**FIGURE 8:**
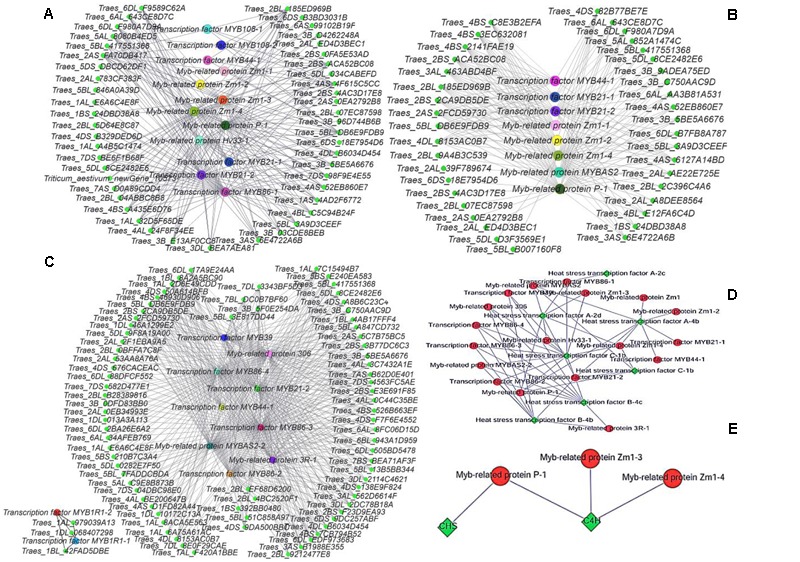
**Gene interaction network diagrams. (A)** Interactions between MYB genes and other genes in the late uninucleate stage. **(B)** Interactions between MYB genes and other genes in the binucleate stage. **(C)** Interactions between MYB genes and other genes in the trinucleate stage. **(D)** Interactions between heat stress genes and other genes. **(E)** Interactions between MYB genes with CHS and C4H genes. In **(A–C)** green represents genes that interact with MYB genes, and each of the other colors represents one type of MYB gene. The protein names are shown for the corresponding genes.

**FIGURE 9:**
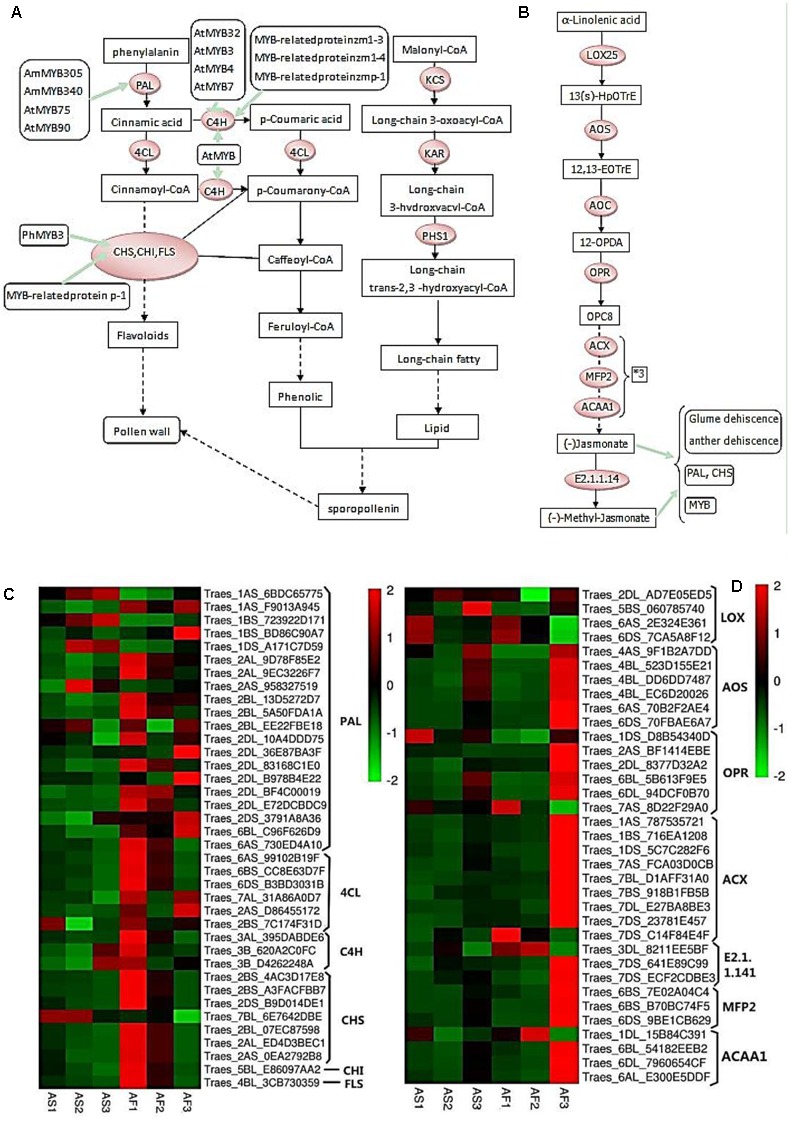
**Phenylpropanoid and jasmonic biosynthetic pathways, and hierarchical clustering of genes encoding related enzymes. (A)** R2R3-MYB transcription factors that regulate the phenylpropanoid biosynthetic pathway in the tapetum and their relationships with pollen wall formation. **(B)** Jasmonate biosynthetic pathway. **(C)** Hierarchical clustering of related genes in the phenylpropanoid biosynthetic pathway. **(D)** Hierarchical clustering of related genes in the jasmonate biosynthetic pathway.

Cytoscape is an open source software platform for visualizing molecular interaction networks and biological pathways, and for integrating these networks with annotations, gene expression profiles and other data (Shannon et al., 2003). In order to obtain a more intuitive understanding of the pivotal roles of MYB transcription factors in the three stages, we selected all of the DEGs with predicted roles as MYB family protein as source nodes and genes that interact with MYB genes as target nodes, before generating an interaction network diagram with Cytoscape (**Figures 8A–C**). In every stage, many genes interacted with MYB genes and one of the main categories comprised by heat stress transcription factors (**Figure 8D**). According to the GO functional annotations and UniProt annotations, most of the MYB genes are linked to jasmonic acid and phenylpropanoid biosynthesis pathways. Interestingly, the KEGG enrichment annotations supported an effect on fertility of the phenylpropanoid biosynthesis pathway, but only between the C4H and CHS genes with MYB related protein p-1, MYB related protein zm1-3, and MYB related protein zm1-4 appeared in the interaction network (**Figure 8E**). Thus, we aimed to analyze the complex relationship among the jasmonic acid and phenylpropanoid biosynthesis pathways, and MYB (**Figures 9A,B**).

As shown in **Figure 9A**, during the pollen maturation process, phenylalanine serves as a substrate for a series of biochemical enzymes, including PAL, C4H, CHS, CHI, and FLS, to form phenolic resins and long chain fatty acids, before finally synthesizing sporopollenin as the main component of the pollen wall (Ouyang and Xue, 1988). The pollen wall is a defensive structure and defects in its structure can lead to abnormal pollen development, which will affect the fertility of plants. The sporopollenin precursor is synthesized in the tapetum and transported to the pollen surface as one of the main components of the pollen wall. As shown in **Figure 8E**, some MYB-related proteins are involved in the phenylpropanoid biosynthetic pathway where they mainly interact with CHS and C4H, and we found that there were increases in the transcription of these genes in AF (**Figure 9C**), thereby ensuring the normal development of pollen. By contrast, AS lacked sufficient expression of these enzymes, thereby affecting the deposition of sporopollenin and pollen wall formation to yield unstable pollen. **Figure 9B** shows that during jasmonic acid synthesis, linolenic acid is released from the cell membrane as the substrate for a series of enzyme reactions. First, alpha-linolenic acid is used as a substrate to synthesize 13(S) – hydrogen peroxide linolenic acid (13-HPOTrE), before allene oxide synthase (AOS) and allene oxide cyclase (AOC) convert 13-HPOT into optically active 12-oxo-phytodienoic acid (12-OPDA). Finally, the effects of 12-OPDA reductase and three consecutive rounds of beta catalytic oxidation generate jasmonic acid. Several genes upstream of the jasmonic acid synthesis pathway are active in the chloroplast, as well as beta-oxidase in the peroxisome, and jasmonic acid is enzymatically modified in the cytoplasm. The synthesis of jasmonic acid and methyl jasmonic acid can regulate the phenylpropanoid biosynthetic pathway by inducing MYB, as well as inducing glume and anther dehiscence, thus more up-regulated genes in fertile AF plants compared with the AS plants during the trinucleate stage to ensure normal fertility (**Figure 9D**).

### Relationships among DEGS in the Phenylpropanoid Biosynthesis Pathway, Jasmonic Acid Biosynthesis Pathway, and MYB Transcription Factors

**FIGURE 10:**
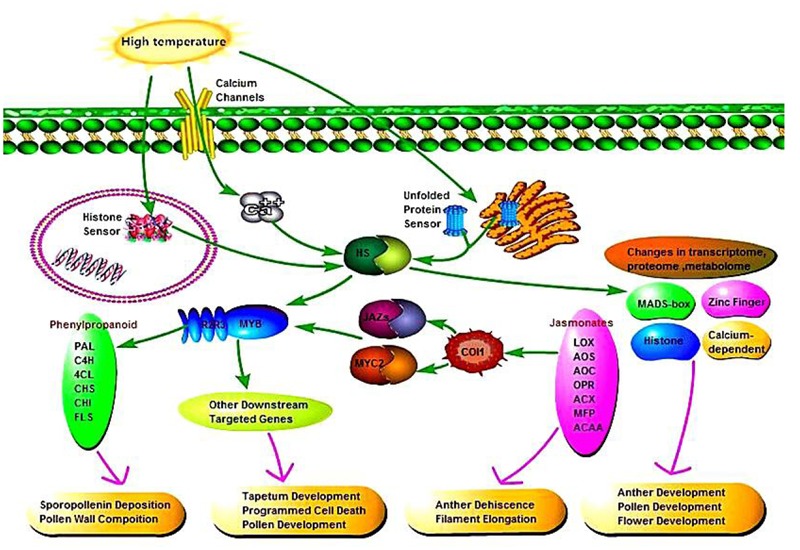
**Signaling pathway connecting phenylpropanoid biosynthesis, jasmonic acid biosynthesis, and MYB transcription factors**.

According to the relationships between phenylpropanoid biosynthesis, jasmonic acid biosynthesis, and MYB transcription factors, we hypothesized a signaling pathway to understand their interactions (**Figure 10**). In plants, at least four putative sensors have recently been proposed to trigger the heat stress response. They include a plasma membrane channel that initiates an inward calcium flux, a histone sensor in the nucleus, and two unfolded protein sensors in the endoplasmic reticulum and the cytosol. Each of these putative sensors is thought to activate heat stress transcription factors leading to changes in transcriptome, proteome and metabolome. High temperature induces the expression of heat stress transcription factors in AF, thereby activating the MYB transcription factors, and jasmonic acid synthesis is also influenced by temperature. Secondary metabolic pathways are induced by MYB as signaling molecules, where phenylpropanoid biosynthesis is affected by jasmonic acid and MYB, thereby affecting pollen outer wall synthesis to ensure the normal pollen development. In addition, jasmonic acid has a physiological function where it affects pollen and glume cracking in the trinucleate stage to make AF fertile, and MYB transcription factors regulate pollen development mainly by affecting the expression of downstream targeted genes.

### qRT-PCR Validation of Partially Candidate Genes

**FIGURE 11:**
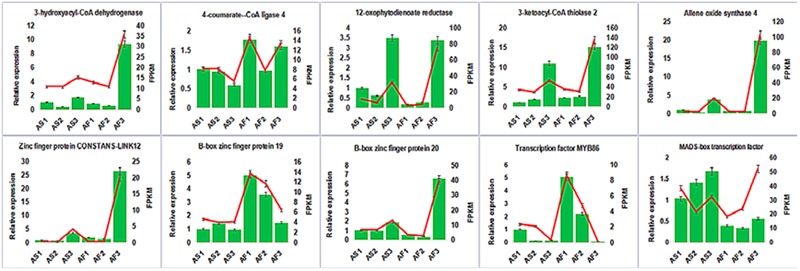
**Candidate DEGs expression levels revealed by qRT-PCR (histogram) and RNA-seq (line chart).** Data from qRT-PCR are means of three replicates and bar represent SE. RPKM from RNA-seq data.

To confirm the accuracy and reproducibility of the transcriptiome analysis result, 10 DEGs were select for qRT-PCR validation. RNA samples from the anther of AS and AF in three stages were used as templates. The expression profiles of the candidate genes revealed by qRT-PCR data were consistent with those derived from sequencing (**Figure 11**). Linear regression analysis of the fold change of the gene expression rations between RNA-seq and qRT-PCR showed positive correlation (Supplemental Figure S6), confirming our transcriptome analysis.

## Discussion

In recent years, the heterosis of wheat has been widely concerned and studied, but due to a variety of factors have not yet cultivated a male sterile material with strong advantage that can be used for large-scale hybrid wheat breeding. The strict temperature and day length requirements for hybrid seed production (Luo et al., 1998), and unstable male sterility (Murai and Tsunewaki, 1993) are key limiting factors in the selection of excellent two-line male sterile wheat materials. KTM3315A as a male sterile wheat material with potential for the production of hybrid wheat, can be employed directly in breeding and hybrid wheat seed production in the winter and spring wheat production zones located in northern China, as well as in the southern autumn-sown spring wheat zone located in Yunnan and Guizhou in China (Meng et al., 2016). Therefore, KTM3315A is worthy of further study of its molecular mechanism of fertility conversion and further improvement.

### GO Annotation

In this study, in terms of cellular components, the DEGs were enriched in the extracellular matrix; evidence is increasing that the plant extracellular matrix can exert a regulatory effect over cell behavior (Roberts, 1994; Cheung and Wu, 1999). For biological processes and molecular functions, the enrichment annotations of DEGs related to circadian rhythm and cell death were similar to those found in previous studies (Zhao et al., 2007). Li et al. (2008) have suggested that the antioxidant activity was higher in fertile lines than sterile lines, which is consistent with our result for the molecular functions of DEGs. The regulation of enzymes is indispensable and thus enzymes must play a key role in fertility conversion, and remains to be further our experiment, when the temperature rises, the extracellular matrix genes will up-regulated expression, which transmit signals to the cells and thus influence expression patterns of intracellular genes.

### Fertility Conversion Candidate Genes Interact with Heat Stress Transcription Factors

According to our analysis using Cytoscape, there are some other candidate fertility conversion genes such as MADS-box transcription factor and zinc finger proteins genes had interactions with heat stress transcription factor genes besides MYB genes (Supplemental Figure S7). The MADS-box transcription factors are important regulatory genes (Shore and Sharrocks, 1995) with a specific conserved domain structure, which can interact with specific DNA sequences to regulate gene expression (Theissen et al., 2000; Theissen and Becker, 2003). In most angiosperms, MADS-box genes are involved in the regulation of flower development (Coen and Meyerowitz, 1991). Zinc finger proteins are transcription factors with a “finger-shaped” structural domain, which is responsible for regulating gene expression (Klug and Rhodes, 1987). More than 30 C_2_H_2_ zinc finger proteins have been found in petunias, including seven zinc finger proteins that are mainly related to anthers, five of which are expressed specifically in anthers (Kapoor et al., 2002). In the present study, the expression levels of MADS-box genes decreased in the initial stages in AF compared with AS, before increasing. However, the expression levels of zinc finger genes were always higher in AF (Supplemental Figure S8 and Table S4). These characteristic trends suggest key roles for these genes in the process of fertility conversion.

### MYB Transcription Factors, Phenylpropanoid Biosynthesis Pathway and Jasmonic Acid Biosynthesis Pathway

MYB transcription factors regulate pollen development mainly by affecting the expression of downstream targeted genes that control tapetum development, programmed cell death (Xu et al., 2014), uninucleate microspore differentiation (Higginson et al., 2003; Steiner-Lange et al., 2003; Zhang Z.B. et al., 2007), pollen spore pigment synthesis, mature pollen function, anther dehiscence, and angiosperm pollen development (Jin et al., 2000; Mu et al., 2015), as well as phenylpropanoid biosynthesis pathway, where they have complementary function, e.g., AtMYB25, AtMYB32, AtMYB4, and AtMYB26 transcription factors control phenylpropanoid metabolism (Preston et al., 2004) and jasmonic acid biosynthesis (Mandaokar and Browse, 2009; Song et al., 2011), as well as being expressed in the anthers to control the deposition of sporopollenin and anther dehiscence. The known roles of partially MYB transcription factors are shown in **Figure 9A**.

It is generally considered that jasmonic acid signaling plays an important role in the regulation of anther dehiscence in *Arabidopsis thaliana* (Mcconn and Browse, 1996; Stintzi and Browse, 2000; Ishiguro et al., 2001; Park et al., 2002; Von Malek et al., 2002). In addition, methyl jasmonic acid induces anther dehiscence *in vitro* in the wheat sterile line BS366 (Qun et al., 2010). These evidences demonstrated that many mutants affected in anther dehiscence are affected in synthesis or sensing of jasmonate. The phenylpropanoids pathway is positively regulated by JA and its derivate methyl jasmonate (MeJA), which have been shown to induce the accumulation of PAL (Kazan and Manners, 2008). The evidence for phenylpropanoids pathway is regulated by JA and methyl jasmonate, was recently explored by a variety of species, such as *Brassica rapa* (Liang et al., 2006) and *Nicotiana tabacum* (Alon et al., 2013), rice (Tianpei et al., 2015).

### Enzymes in the Phenylpropanoid and Jasmonic Acid Biosynthetic Pathways

PAL, C4H, 4CL, CHS, and CHI are the main enzymes involved in phenylpropanoid synthesis. PAL is a rate-limiting enzyme in the first step of phenylpropanoid synthesis (Li et al., 2007). C4H and 4CL are critical enzymes for the catalytic synthesis of the phenolic precursor 4-coumaric acid-CoA. CHS catalyzes the synthesis of chalcones, which are important precursors of flavones. Thus, the genes for these enzymes were upregulated during the three stages in AF compared with AS (**Figure 9C**). The phenylpropanoid synthesis pathway is complex and it generates various products, which suggests that the upregulated expression of these genes yields flavonoids, increased sporopollenin synthesis, and a smooth pollen outer wall, and the increased synthesis of other metabolic substances may also be beneficial for the normal development of pollen. Lipoxygenase (LOX), AOS, AOC, and OPR are key enzymes in the jasmonic acid synthesis pathway, and although the LOX gene was downregulated in the late uninucleate stage, the other genes were upregulated in the late uninucleate and trinucleate stages, but they were not differentially expressed in the binucleate stage. Therefore, although the binucleate stage is crucial for the determination of fertility, jasmonic acid synthesis was not significantly different in AF compared with the AS in this stage.

### MYB-Related Protein p-1

**FIGURE 12:**
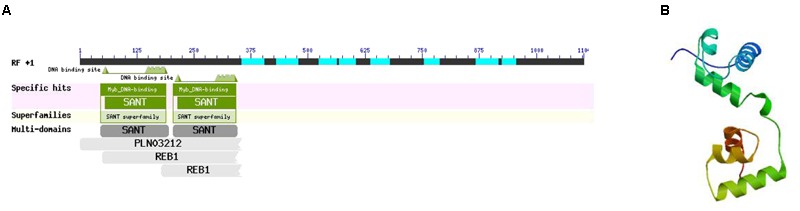
**Structural domain (A)** and tertiary structure **(B)** of MYB-related protein p-1.

According to the interaction network, there was a specific relationship between MYB-related protein p-1 and CHS. GO analysis determined that the gene cellular component was nucleus (GO: 0005634) and the biological processes included sequence-specific DNA binding transcription factor activity (GO: 0003700), response to jasmonic acid (GO: 0009753), cinnamic acid biosynthetic process (GO: 0009800), coumarin biosynthetic process (GO: 0009805), and regulation of phenylpropanoid metabolic process (GO: 2000762). Thus, the GO classification showed that MYB-related protein p-1 participates in the phenylpropanoid and jasmonic acid biosynthesis pathways. In order to further explore its specific physical and chemical properties and structure, we predicted the amino acid sequence using ExPASy, which we then analyzed using ProtParam, and the structural domain was BLASTed via the NCBI website. MYB-related protein p-1 comprises 367 amino acids (molecular weight = 39334.1, theoretical PI = 4.84, formula = C1704H2698N472O558S19) and like R2R3-MYB, it has two DNA binding sites (**Figure 12A**). We predicted its tertiary structure using SWISS–MODEL, which identified six helical structures (**Figure 12B**).

## Conclusion

The K-TCMS system is used for hybrid breeding in wheat (*Triticum aestivum* L.), and it could also be employed for hybrid wheat seed multiplication and production. The K-TCMS line is crossed with a pollinator line during the normal wheat-growing season to produce hybrid wheat seeds, whereas the TCMS line is maintained by self-pollination in a high-temperature environment. However, the mechanism responsible for the male sterility/fertility transition still needs to be determined in K-TCMS. To elucidate the molecular mechanism of fertility conversion in TCMS wheat, we treated KTM 3315A in an artificial climate box at temperatures above and below the fertility conversion threshold temperature. The anthers obtained before and after the critical stage for fertility conversion (binucleate stage) were sequenced by RNA-Seq and verified by qPCR. Using the sequencing data, we employed GO, COG, and KEGG classifications to annotate the DEGs. Our analysis showed that the fertility conversion mechanism in K-TCMS wheat mainly involves the phenylpropanoid biosynthesis pathway, jasmonate biosynthesis pathway, and MYB transcription factors in the fertility conversion stage. In addition, some DEGs that interact with heat stress transcription factors are associated with fertility conversion in K-TCMS. Thus, we obtained candidate genes related to pollen development and fertility conversion in K-TCMS wheat, and we highlighted the key involvement of two biosynthesis networks in anther development. Our results demonstrate that male sterility/fertility is not likely to be attributable to the regulation of a single gene, but instead many genes participate in this complex regulatory mechanism. Our results will contribute to the improved use of two-line hybrids and enhance the processes employed for hybrid wheat utilization and research.

## Author Contributions

XS, JY conceived the original screening and research plans; XS and LZ supervised the experiments; JY performed most of the experiments, analyzed the data and prepared the figures and tables; YD, PY, ZL, XG, and GZ provided technical assistance to JY; LZ, GH, advised on the analysis and interpretation of the results. JY wrote the article with contributions of all the authors. All authors approved the final manuscript.

## Conflict of Interest Statement

The authors declare that the research was conducted in the absence of any commercial or financial relationships that could be construed as a potential conflict of interest.

## Footnotes

1 https://urgi.versailles.inra.fr/download/iwgsc

2 http://www.omicshare.com/tools/index

3 http://web.expasy.org/translate/

4 http://web.expasy.org/protparam/

5 https://blast.ncbi.nlm.nih.gov/Blast.cgi?PROGRAM=blastx&PAGE_TYPE=BlastSearch&LINK_LOC=blasthome

6 https://swissmodel.expasy.org/interactive#sequence

7 https://www.ncbi.nlm.nih.gov/tools/primer-blast/

8 http://www.expasy.ch/sprot
