# Supplementary material for: Identification of Candidate Genes and Biosynthesis Pathways Related to Fertility Conversion by Wheat KTM3315A Transcriptome Profiling
Source: Front Plant Sci. 2017 Apr 6;8:449. doi: 10.3389/fpls.2017.00449 (PMC5382222; doi:10.3389/fpls.2017.00449)
Supplement: Supplementary file 1 [file Table_1.DOCX]

**Supplemental Table S1**. Evaluation statistics of sample sequencing data

| ID | Total Reads | GC (%) | N (%) | Q30 (%) | Mapped Reads | Uniq Mapped Reads |
| --- | --- | --- | --- | --- | --- | --- |
| AS1 | 102375824 | 55.08 | 0.00 | 96.05 | 71138054(69.49%) | 59717986(83.95%) |
| AS2 | 98979736 | 53.89 | 0.00 | 96.01 | 67846287(68.55%) | 56874196(83.83%) |
| AS3 | 99577808 | 54.06 | 0.00 | 95.52 | 68154087(68.44%) | 57023290(83.67%) |
| AF1 | 91760042 | 54.10 | 0.00 | 95.92 | 64703661(70.51%) | 53606244(82.85%) |
| AF2 | 86152608 | 54.53 | 0.00 | 96.07 | 59739244(69.34%) | 50544487(84.61%) |
| AF3 | 100087256 | 54.48 | 0.00 | 95.76 | 65905470(65.85%) | 55679128(84.48%) |
