# Supplementary material for: Identification of Candidate Genes and Biosynthesis Pathways Related to Fertility Conversion by Wheat KTM3315A Transcriptome Profiling
Source: Front Plant Sci. 2017 Apr 6;8:449. doi: 10.3389/fpls.2017.00449 (PMC5382222; doi:10.3389/fpls.2017.00449)
Supplement: Supplementary file 5 [file Presentation_1.PDF]

Supplemental Figure S1

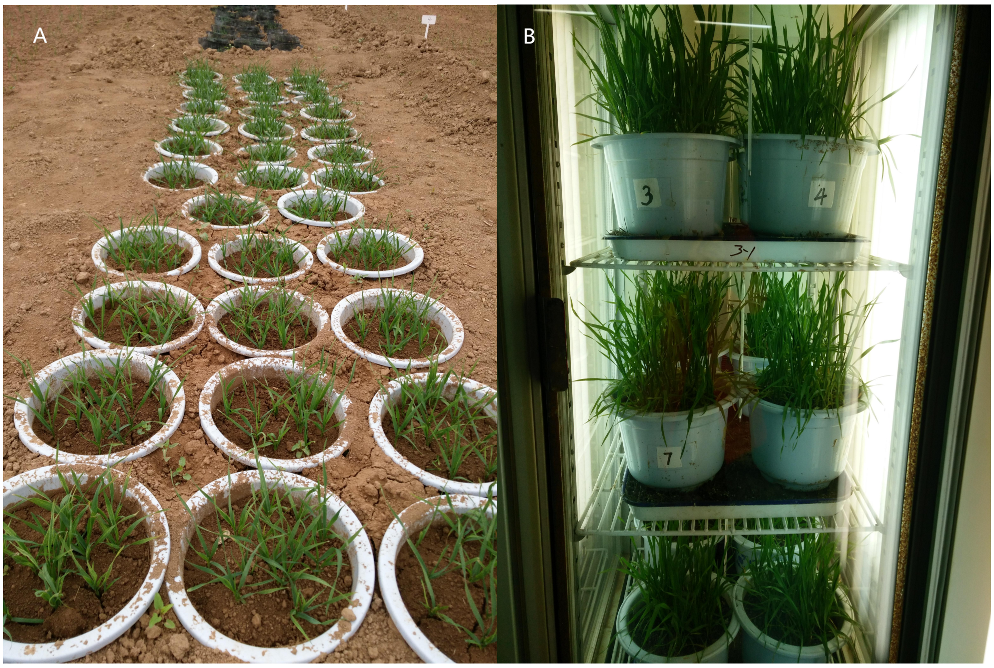

Fig S1. KTM3315A plants grown in the field were transferred into flower pots filled with arable field clay(A);KTM3315A were grown in artificial climate box under different temperature-controlled treatments(B).

Supplemental Figure S2

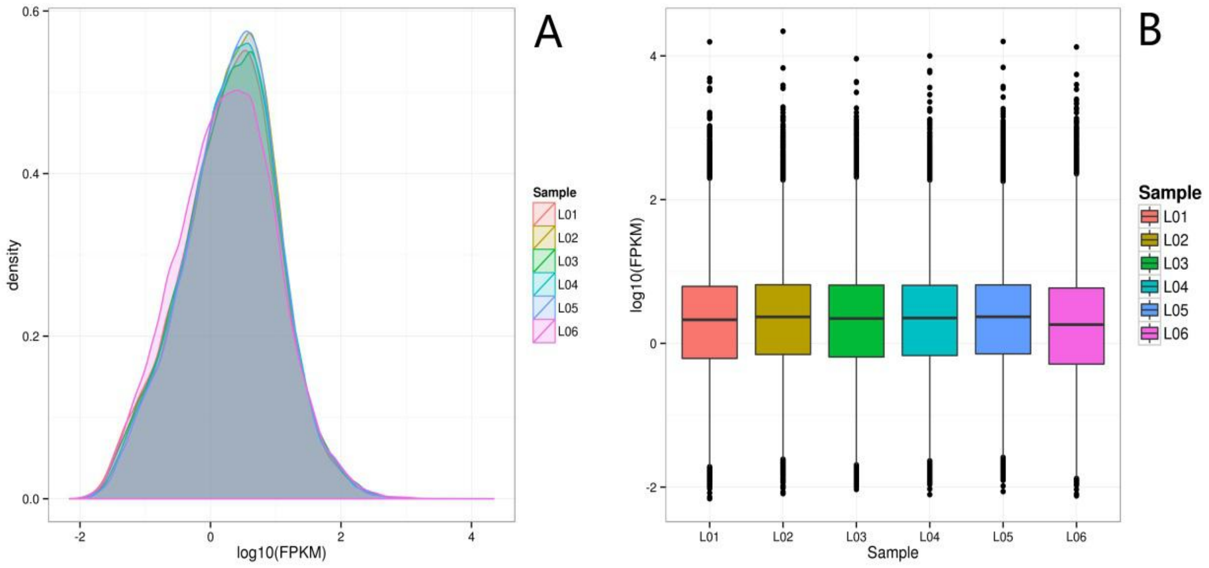

Fig S2. FPKM density (A) and boxplot FPKM distribution (B) each sample. (A) The different color curve presents different samples, the horizontal axis indicates corresponding sample FPKM, The vertical axis indicates corresponding probability density; (B) The horizontal axis represents different samples, the vertical axis represents samples FPKM expression quantity of value. AS, L01-L03; AF, L04-L06. L01, L04, the later uninucleate stage； L02, L05, the binucleate stage； L03, L06, the trinucleate stage.

Supplemental Figure S3

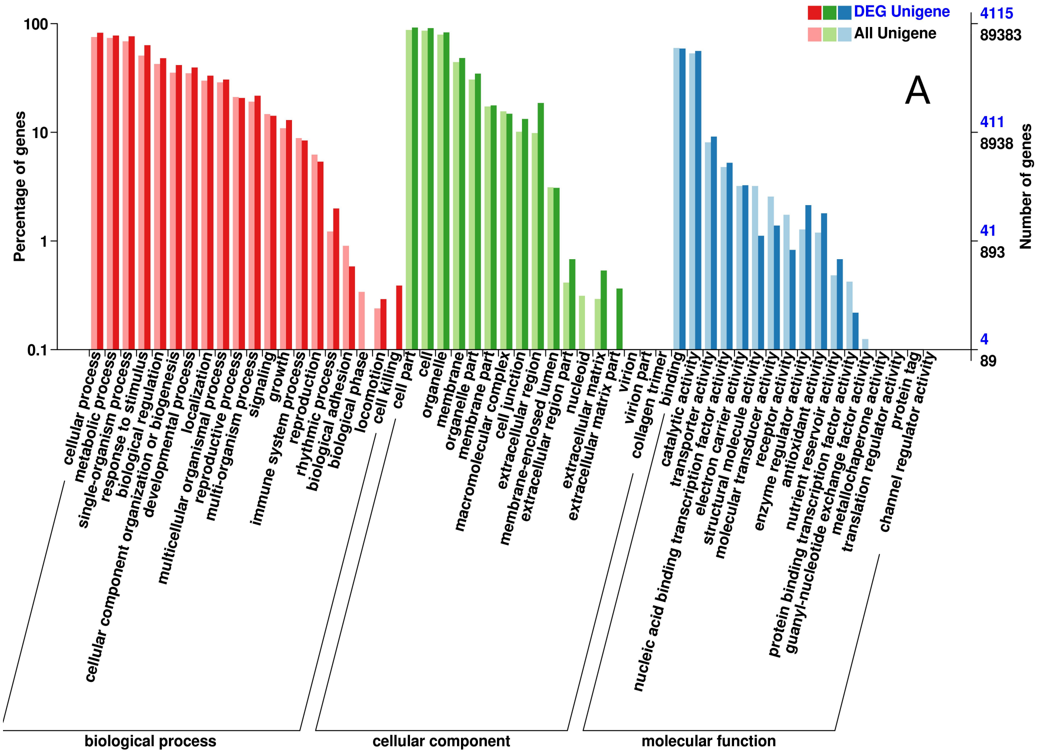

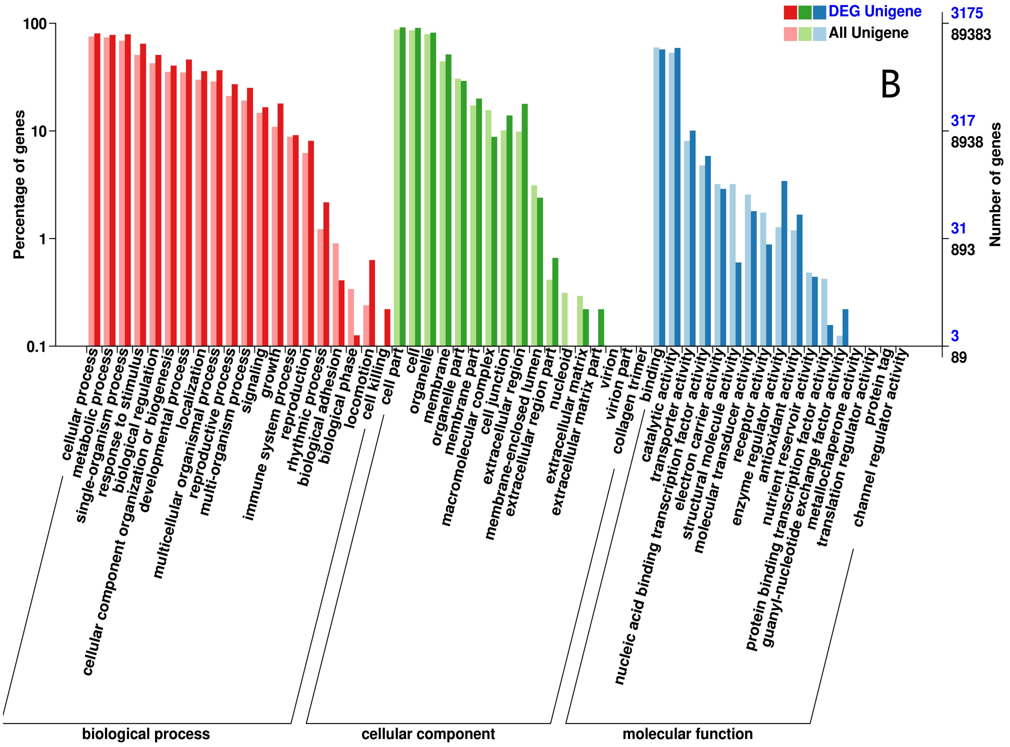

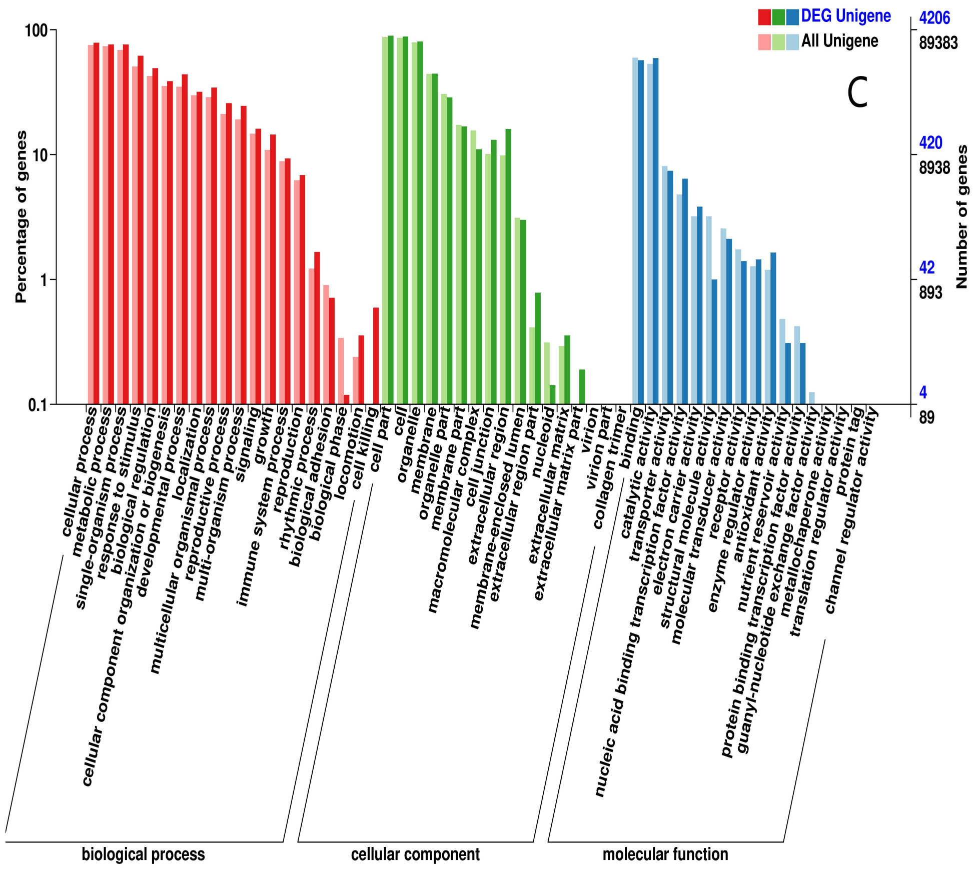

Supplemental Figure S3. Classification statistics for differentially expressed genes according to the GO annotations. (A), (B) and (C) represent theGO annotation classification statistics for differentially expressed genes in AF relative to AS in the late uninucleate,binucleate and trinucleate stages, respectively.

Supplemental Figure S4

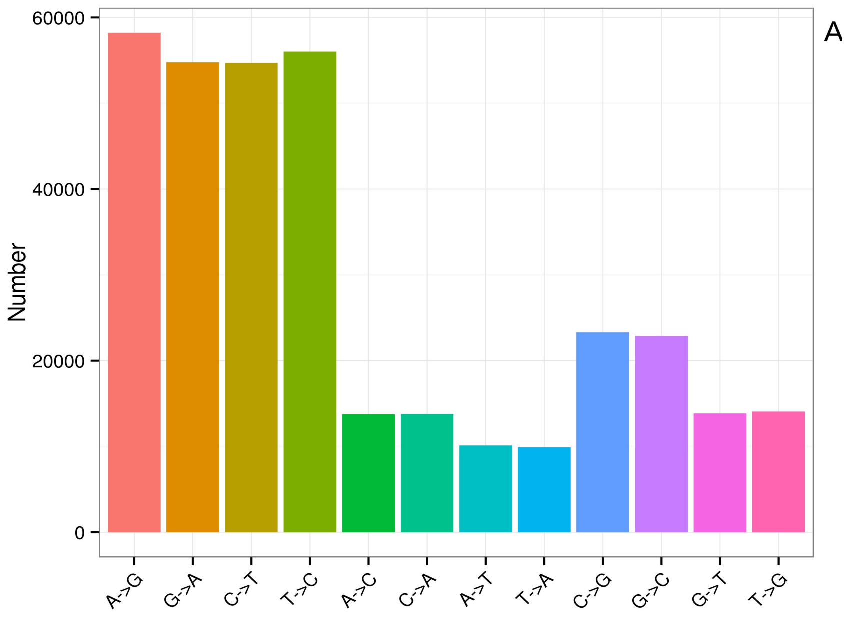

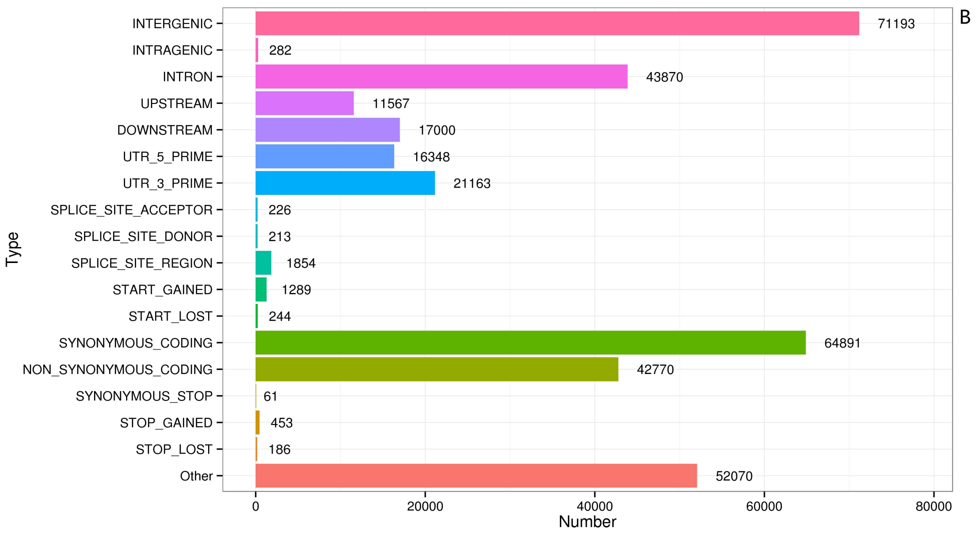

Fig S4. SNP mutation type statistical distribution (A) and SNP annotation statistic (B).The horizontal axis is the SNP mutation type, and the vertical axis is the corresponding SNP number (A).The vertical axis is the region or type of SNP, and the horizontal axis is the number of categories(B).

Supplemental Figure S5

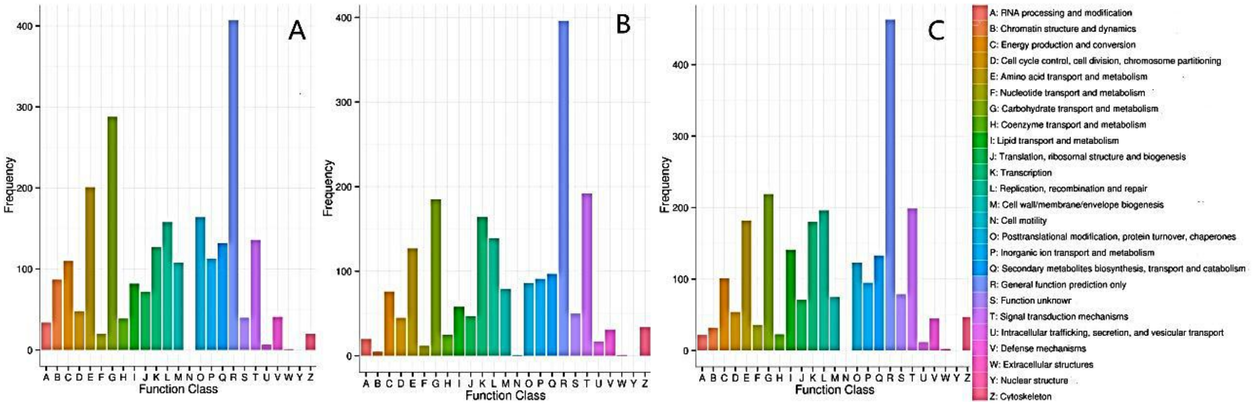

Fig S5. Classification statistics of differentially expressed genes COG annotation. (A), (B), (C) respectively represent COG annotation classification statistics of differentially expressed genes in AF contrast to AS in three different stages (the late uninucleate stage, the binucleate stage, and the trinucleate stage).

Supplemental Figure S6

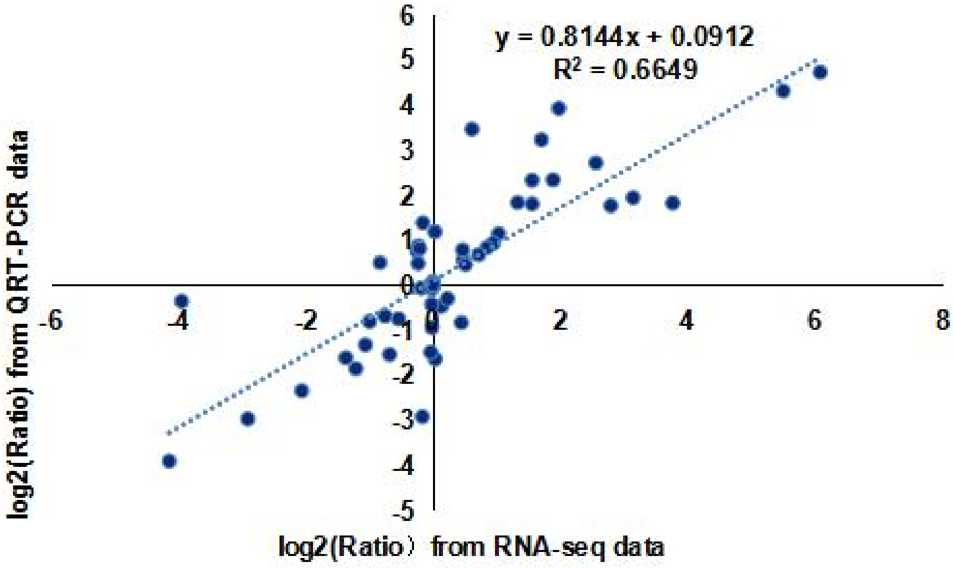

Fig S6. Coefficient analysis of fold change data between qRT-PCR and RNA-seq. Data indicating relative transcript level from qRT-PCR are means of three replicates .RPKM from RNA-seq data. Scatterplots were generate by log2 expression ration from RNA-seq (x-axis) and qRT-PCR (y-axis).

Supplemental Figure S7

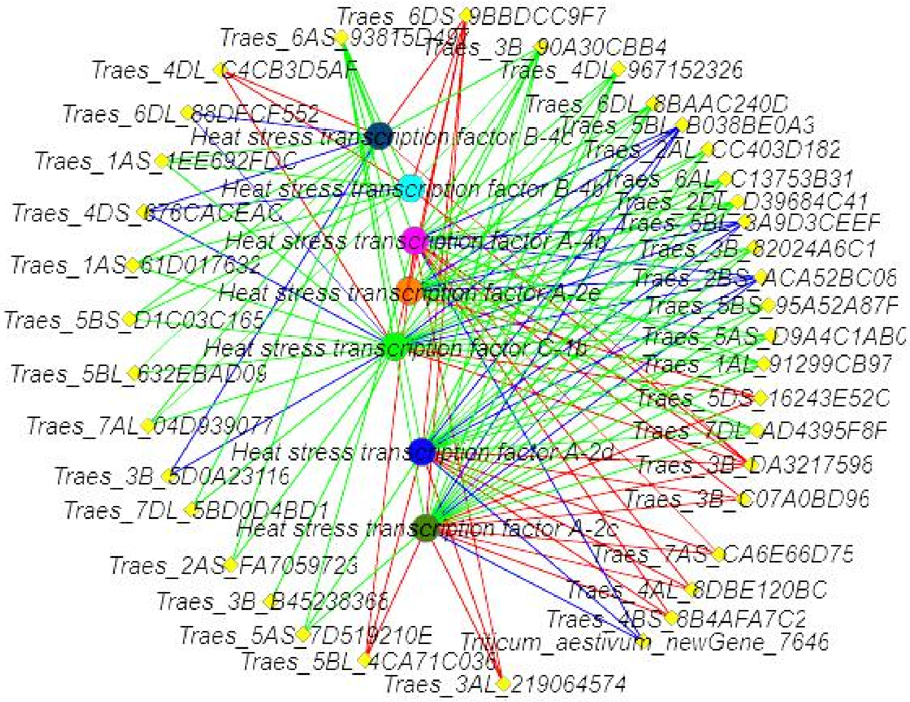

Fig S7 Interact network diagram of heat stress transcription factor genes and other genes. different colors circle represents different kinds of heat stress transcription factor genes, yellow rhombus represents various genes that interact with heat stress transcription factor genes; red, blue and green lines represent the interactions between the heat stress transcription factor genes and AD-BOX，zinc-finger and MYB genes.

Supplemental Figure S8

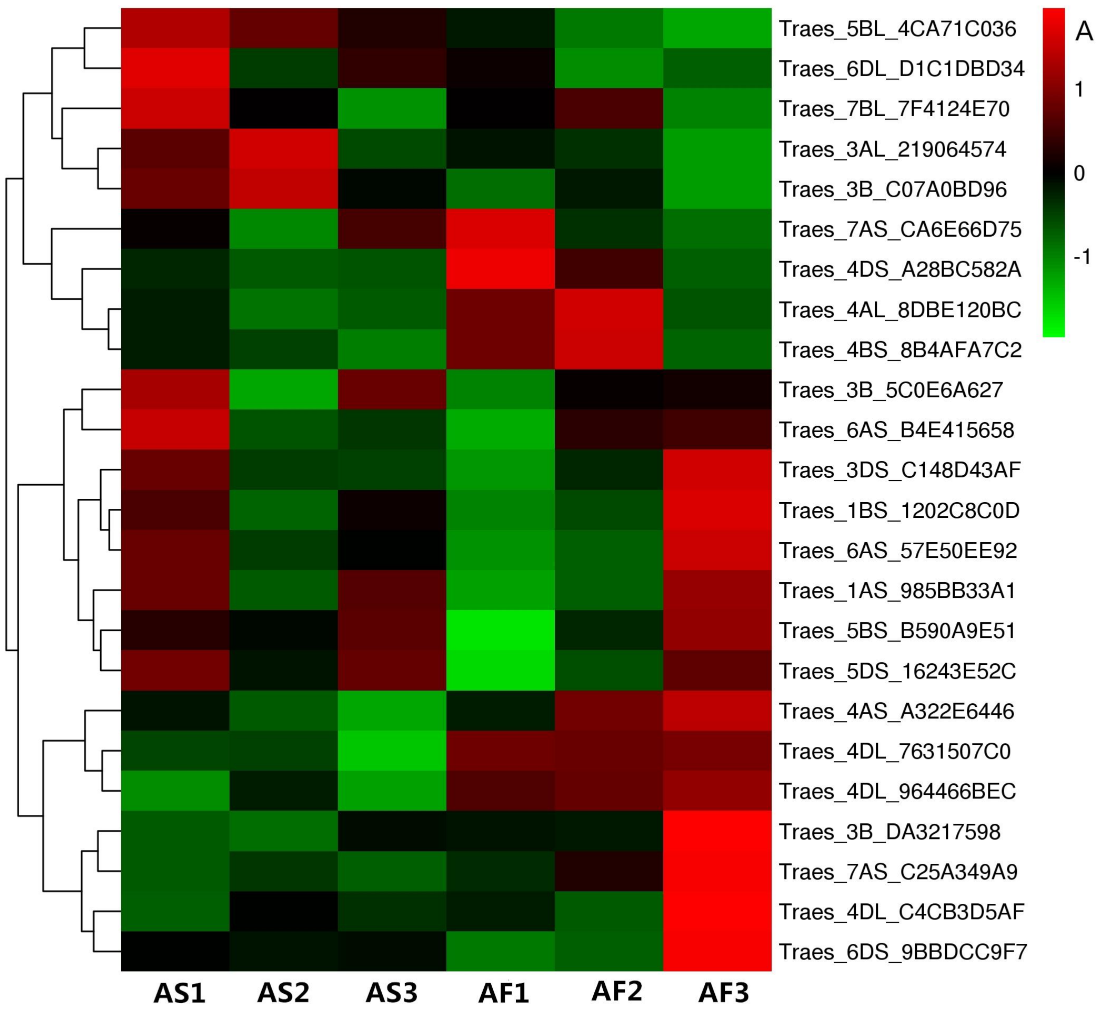

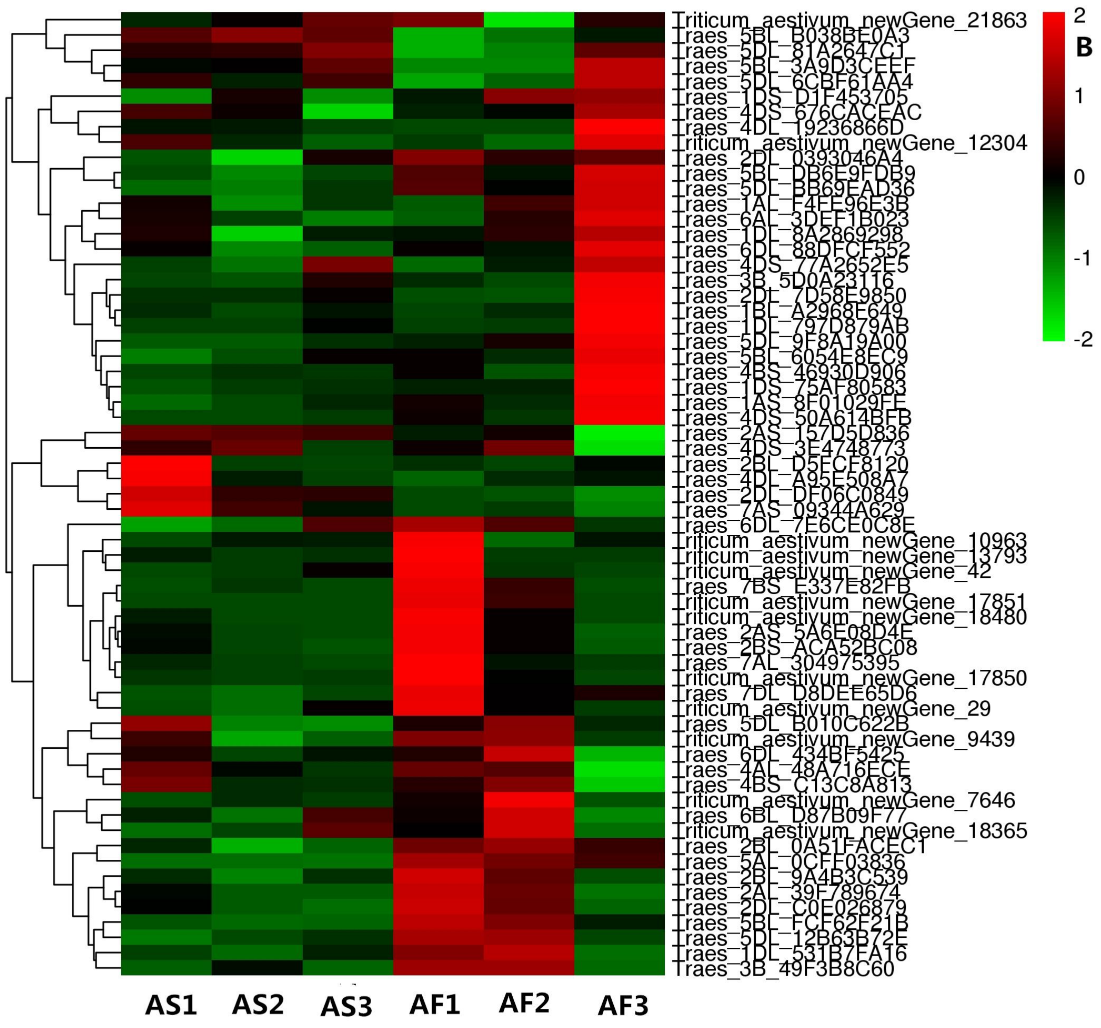

Fig S8. Hierarchical clustering of related genes encoding MADS-box transcription factor(A) and Zinc finger protein(B).

Supplemental Table S1. Evaluation statistics of sample sequencing data

ID	Total Reads	GC (%)	N (%)	Q30 (%)	Mapped Reads	Uniq Mapped Reads
AS1	102375824	55.08	0.00	96.05	71138054(69.49%)	59717986(83.95%)
AS2	98979736	53.89	0.00	96.01	67846287(68.55%)	56874196(83.83%)
AS3	99577808	54.06	0.00	95.52	68154087(68.44%)	57023290(83.67%)
AF1	91760042	54.10	0.00	95.92	64703661(70.51%)	53606244(82.85%)
AF2	86152608	54.53	0.00	96.07	59739244(69.34%)	50544487(84.61%)
AF3	100087256	54.48	0.00	95.76	65905470(65.85%)	55679128(84.48%)

Supplemental Table S2. Sequence-specific primers used for qRT-PCR.

Primer name	Sequence(5'to3')	Annotion
Traes_1BS_1202C8C0D-F	GGCTACTACTGGGTGGGGA	MADS-box transcription factor 58
Traes_1BS_1202C8C0D-R	CTCCTTGGTGGTGCCTTTCT	
Traes_5BL_FCF62F21B-F	GGGCTTGCGGACCCTAATAA	B-box zinc finger protein 19
Traes_5BL_FCF62F21B-R	CATGGGTTCGTTTCCCACCA	
Traes_2DL_7D58E9850-F	TCCGAGTACCTGACGAAGAC	B-box zinc finger protein 20
Traes_2DL_7D58E9850-R	ACCCTGCTGATACGGCTTG	
Traes_1DL_797D879AB-F	GGCTACTCATACTCCCCCACA	Zinc finger protein CONSTANS-LIKE 12
Traes_1DL_797D879AB-R	CGAGGTGGTGGCAGATGTTG	
Traes_6DL_94DCF0B70-F	TTATCCAACCCGGACTTGCC	12-oxophytodienoate reductase
Traes_6DL_94DCF0B70-R	CGGACGGTAGAAACGGGTAG	
Traes_4AL_DACD935B2-F	GAGCAGTGGAACAACCTCGT	Transcription factor MYB86
Traes_4AL_DACD935B2-R	TGGTGCCCGACTTGGTAGTA	
Traes_7AL_31A86A0D7-F	TTCTCATCACACACGCCGAA	4-coumarate--CoA ligase 4
Traes_7AL_31A86A0D7-R	GGAACCGAGTCAGCGAAGAA	
Traes_6DS_70FBAE6A7-F	GGTCGGGAGGCTAATGGTG	Allene oxide synthase 4
Traes_6DS_70FBAE6A7-R	GCTCTAGCGGCATCTCCTTC	
Traes_6BS_7E02A04C4-F	AAGTACAGGGGTGGCCTCAT	3-hydroxyacyl-CoA dehydrogenase
Traes_6BS_7E02A04C4-R	ACAAAACAAGACAGTGGTCGG	
Traes_6DL_7960654CF-R	GCCTTATCTTGCTCTTCTCTGG	3-ketoacyl-CoA thiolase 2
Traes_6DL_7960654CF-F	TCGGTGAACTCTGTTGGTTG	
Actin-F	CTCCCTCACAACAACCGC	Actin
Actin-R	TACCAGGAACTTCCATACCAAC	

Supplemental Table S3. The mainly enriched KEGG pathways cluster of DEGs in the three stages.

	Kegg_pathway	ko_id	Cluter_frequency	Genome_frequency	P-value	Corrected_P-value
**AF1vsAS1**	Starch and sucrose metabolism	ko00500	141 out of 1154 12.2183708838821%	893 out of 22706 3.93288117678147%	0	0
	Phenylpropanoid biosynthesis	ko00940	115 out of 1154 9.96533795493934%	1097 out of 22706 4.83132211750198%	0	0
	Galactose metabolism	ko00052	54 out of 1154 4.67937608318891%	402 out of 22706 1.77045714789043%	7.14E-11	8.07E-09
	Cyanoamino acid metabolism	ko00460	41 out of 1154 3.5528596187175%	298 out of 22706 1.3124284330133%	6.75E-09	7.63E-07
	Carbon metabolism	ko01200	100 out of 1154 8.66551126516464%	1085 out of 22706 4.77847265040077%	5.14E-09	5.81E-07
	Cysteine and methionine metabolism	ko00270	46 out of 1154 3.98613518197574%	392 out of 22706 1.72641592530609%	1.19E-07	1.34E-05
	Arginine and proline metabolism	ko00330	30 out of 1154 2.59965337954939%	294 out of 22706 1.29481194397956%	0.0002362	0.026687897
	beta-Alanine metabolism	ko00410	18 out of 1154 1.55979202772964%	164 out of 22706 0.722276050383159%	0.0017556	0.198385132
	Circadian rhythm - plant	ko04712	17 out of 1154 1.47313691507799%	161 out of 22706 0.709063683607857%	0.0034833	0.393617482
**AF2vsAS2**	Starch and sucrose metabolism	ko00500	101 out of 842 11.9952494061758%	893 out of 22706 3.93288117678147%	4.70E-12	5.31E-10

	Phenylpropanoid biosynthesis	ko00940	87 out of 842 10.332541567696%	1097 out of 22706 4.83132211750198%	1.52E-11	1.72E-09
	Galactose metabolism	ko00052	41 out of 842 4.86935866983373%	402 out of 22706 1.77045714789043%	5.39E-09	6.09E-07
	Cyanoamino acid metabolism	ko00460	26 out of 842 3.08788598574822%	298 out of 22706 1.3124284330133%	5.23E-05	5.91E-03
	Carbon metabolism	ko01200	59 out of 842 7.00712589073634%	1085 out of 22706 4.77847265040077%	2.17E-03	2.45E-01
	Cysteine and methionine metabolism	ko00270	34 out of 842 4.03800475059382%	392 out of 22706 1.72641592530609%	4.45E-06	0.000503193
	Arginine and proline metabolism	ko00330	20 out of 842 2.37529691211401%	294 out of 22706 1.29481194397956%	0.0069898	0.789850266
	beta-Alanine metabolism	ko00410	18 out of 842 2.13776722090261%	164 out of 22706 0.722276050383159%	3.99E-05	0.004513456
	Circadian rhythm - plant	ko04712	22 out of 842 2.61282660332542%	161 out of 22706 0.709063683607857%	1.36E-07	1.54E-05
**AF3vsAS3**	Starch and sucrose metabolism	ko00500	86 out of 1013 8.48963474827246%	893 out of 22706 3.93288117678147%	2.58E-12	3.04E-10
	Phenylpropanoid biosynthesis	ko00940	99 out of 1013 9.77295162882527%	1097 out of 22706 4.83132211750198%	4.32E-13	5.09E-11
	Galactose metabolism	ko00052	57 out of 1013 5.62685093780849%	402 out of 22706 1.77045714789043%	1.88E-12	2.21E-10
	Cyanoamino acid metabolism	ko00460	28 out of 1013 2.76406712734452%	298 out of 22706 1.3124284330133%	0.0001778	0.020974748

Carbon metabolism	ko01200	71 out of 1013 7.00888450148075%	1085 out of 22706 4.77847265040077%	0.0008022	0.09465993
Cysteine and methionine metabolism	ko00270	30 out of 1013 2.96150049358342%	392 out of 22706 1.72641592530609%	0.0030096	0.355132082
Arginine and proline metabolism	ko00330	23 out of 1013 2.27048371174729%	294 out of 22706 1.29481194397956%	0.0067126	0.792088116
beta-Alanine metabolism	ko00410	16 out of 1013 1.57946692991116%	164 out of 22706 0.722276050383159%	2.79E-03	3.30E-01
Circadian rhythm - plant	ko04712	18 out of 1013 1.77690029615005%	161 out of 22706 0.709063683607857%	3.17E-04	3.74E-02
